# Proteomics and secreted lipidomics of mouse-derived bone marrow cells exposed to a lethal level of ionizing radiation

**DOI:** 10.1038/s41598-023-35924-9

**Published:** 2023-05-31

**Authors:** Yota Tatara, Satoru Monzen

**Affiliations:** 1 Department of Stress Response Science, Center for Advanced Medical Research, Graduate School of Medicine, Hirosaki University, 5 Zaifu-Cho, Hirosaki, Aomori, 036-8562 Japan; 2 Department of Radiation Science, Graduate School of Health Sciences, Hirosaki University, 66-1 Hon-Cho, Hirosaki, Aomori, 036-8564 Japan

**Keywords:** Prognostic markers, Proteomics, Lipidomics

## Abstract

High doses of ionizing radiation (IR) exposure can lead to the development of severe acute radiation syndrome with bone marrow failure. Defining risk factors that predict adverse events is a critical mission to guide patient selection for personalized treatment protocols. Since non-hematopoietic stem cells act as feeder cells in the niche and their secreted lipids may regulate hematopoietic stem cells, we focused on non-hematopoietic stem cells and aimed to discover biomarkers that can assess radiation exposure from their secreted lipids. Bone marrow stromal cells (BMSCs) and osteoblast differentiation-inducing cells (ODICs) isolated from mouse femurs were exposed to lethal doses of IR and the proteomic differences between BMSC and ODIC cell layers were compared. We observed an increased Nrf2-mediated oxidative stress response and IL6 expression in ODICs and decreased expression of mitochondrial proteins in BMSCs. To elucidate secreted factors, lipidomics of the cultures were profiled; the relevant lipids distinguishing IR-exposed and control groups of BMSC were acyl-acyl phosphatidylcholine (PC aa C34:1 and PC aa C34:4), lysophosphatidylcholine (lyso-PC a C18:0 and lyso PC a C17:0) and sphingomyelin (SM C20:2). These analyses suggest that certain lipids are candidate markers for the toxic effects of IR.

## Introduction

Acute radiation syndrome (ARS) is caused by exposure to high doses of ionizing radiation (IR), whether due to an accident or a deliberate terrorist attack with the use of radiation sources. ARS involves bone marrow and gastrointestinal necrosis and often leads to multi-organ disease syndrome^1,2^. The treatment success of ARS depends on the treatment onset time^3–5^. The earlier symptoms of ARS occur during the prodromal phase (< 48 h after exposure) following the latent phase (2–20 days after exposure) and the fully developed phase (21–60 days after exposure)^6^. For preventing infections, excessive immune responses, and tissue breakdown caused by ARS, treatment with antibiotics, cytokines, blood transfusion, and/or stem cell transplantation can be supplied. The prediction of the dose absorbed by a victim is required for the treatment and possible prevention of ARS^6^. The chromosomal aberration assay in peripheral blood lymphocytes is the gold standard in biological dosimetry^7,8^. However, there are some concerns to this assay^9^: (1) The doses of IR in the body are often inhomogeneous. (2) Repair of the chromosomal aberration intervenes in the estimation of the dose. (3) Lymphocytes carrying unstable aberrations disappear relatively rapidly. It limits retrospective dosimetry. Therefore, the assay is only possible because of blood collection within hours before the event of leukopenia^7^. In addition to those, the chromosomal aberration assay has a disadvantage that takes time to culture lymphocytes^9^. Therefore, a novel diagnosis that allow for prompt triage of patients with a risk of developing ARS is desired^10^.

Hematopoietic and mesenchymal stem cells in the bone marrow produce autocrine and paracrine secretory factors (the secretome) that play critical roles in maintaining an undifferentiated state and regulating the proliferation and differentiation of stem cells^11^. Some reports have shown that secreted lipid mediators affect bone metabolism and differentiation^11–14^. However, the set of secreted lipids, known as the lipidome, of the bone marrow stromal cells and the stem cell niche has not been investigated in detail. Cellular exposure to IR leads to oxidizing events that directly alter the atomic structure of target macromolecules such as lipids, proteins, and nucleic acids; these changes can also occur via products of water radiolysis^15^. Reactive oxygen species (ROS) react readily with lipids and can initiate the lipid peroxidation process, a chain reaction that produces multiple breakdown molecules, such as malonaldehyde and 4-hydroxyalkenals^16^. Therefore, investigation of the effects of IR on lipid metabolism may lead to the identification of one or more secretory factors that reflect the biological response to IR, and consequently, result in identifying a risk factor for ARS exacerbation.

To clarify the association with IR experiments on individual mice or bone tissue in the future, we employed an irradiated model using mouse bone marrow-derived stromal cells (BMSC) and osteoblastic differentiation-induced cells (ODIC) in this study. We investigated the secreted lipidome of BMSC and ODIC exposed to IR, targeting lipids as listed in Table 1 and Supplemental Table S1. Furthermore, we also performed a proteomic approach to examine the effects of IR on the cells from a functional viewpoint. Based on these analyses, we attempted to elucidate an overview of the impact of IR on bone marrow stromal cells and osteoblasts, which form the hematopoietic stem cell niche^17^, and aimed to discover biomarkers for assessing radiation exposure by secreted lipids.

**Table 1:** List of targeted lipids.

Lipid class	Number of analytes	Analyte abbreviation
Sphingomyeline, Hydroxysphingomyelins	15	SM (OH) C14:1, SM (OH) C16:1, SM (OH) C22:1, SM (OH) C22:2, SM (OH) C24:1, SM C16:0, SM C16:1, SM C18:0, SM C18:1, SM C20:2, SM C22:3, SM C24:0, SM C24:1, SM C26:0, SM C26:1
Diacyl phosphatidylcholine	38	PC aa C24:0, PC aa C26:0, PC aa C28:1, PC aa C30:0, PC aa C30:2, PC aa C32:0, PC aa C32:1, PC aa C32:2, PC aa C32:3, PC aa C34:1, PC aa C34:2, PC aa C34:3, PC aa C34:4, PC aa C36:0, PC aa C36:1, PC aa C36:2, PC aa C36:3, PC aa C36:4, PC aa C36:5, PC aa C36:6, PC aa C38:0, PC aa C38:1, PC aa C38:3, PC aa C38:4, PC aa C38:5, PC aa C38:6, PC aa C40:1, PC aa C40:2, PC aa C40:3, PC aa C40:4, PC aa C40:5, PC aa C40:6, PC aa C42:0, PC aa C42:1, PC aa C42:2, PC aa C42:4, PC aa C42:5, PC aa C42:6
Acyl-alkyl phosphatidylcholine	38	PC ae C30:0, PC ae C30:1, PC ae C30:2, PC ae C32:1, PC ae C32:2, PC ae C34:0, PC ae C34:1, PC ae C34:2, PC ae C34:3, PC ae C36:0, PC ae C36:1, PC ae C36:2, PC ae C36:3, PC ae C36:4, PC ae C36:5, PC ae C38:0, PC ae C38:1, PC ae C38:2, PC ae C38:3, PC ae C38:4, PC ae C38:5, PC ae C38:6, PC ae C40:1, PC ae C40:2, PC ae C40:3, PC ae C40:4, PC ae C40:5, PC ae C40:6, PC ae C42:0, PC ae C42:1, PC ae C42:2, PC ae C42:3, PC ae C42:4, PC ae C42:5, PC ae C44:3, PC ae C44:4, PC ae C44:5, PC ae C44:6
Lysophosphatidylcholine	14	lysoPC a C14:0, lysoPC a C16:0, lysoPC a C16:1, lysoPC a C17:0, lysoPC a C18:0, lysoPC a C18:1, lysoPC a C18:2, lysoPC a C20:3, lysoPC a C20:4, lysoPC a C24:0, lysoPC a C26:0, lysoPC a C26:1, lysoPC a C28:0, lysoPC a C28:1

Abbreviations are as follows: Cx:y: (x = number of carbons in the fatty acid side chain, y = number of double bonds in the fatty acid side chain), OH: hydroxyl, PC: phosphatidylcholine, aa: acyl‐acyl (aa), ae: acyl‐alkyl, a: lyso, SM: sphingomyelin.

## Results

### Characterization of BMSC and ODIC

**Figure 1:**
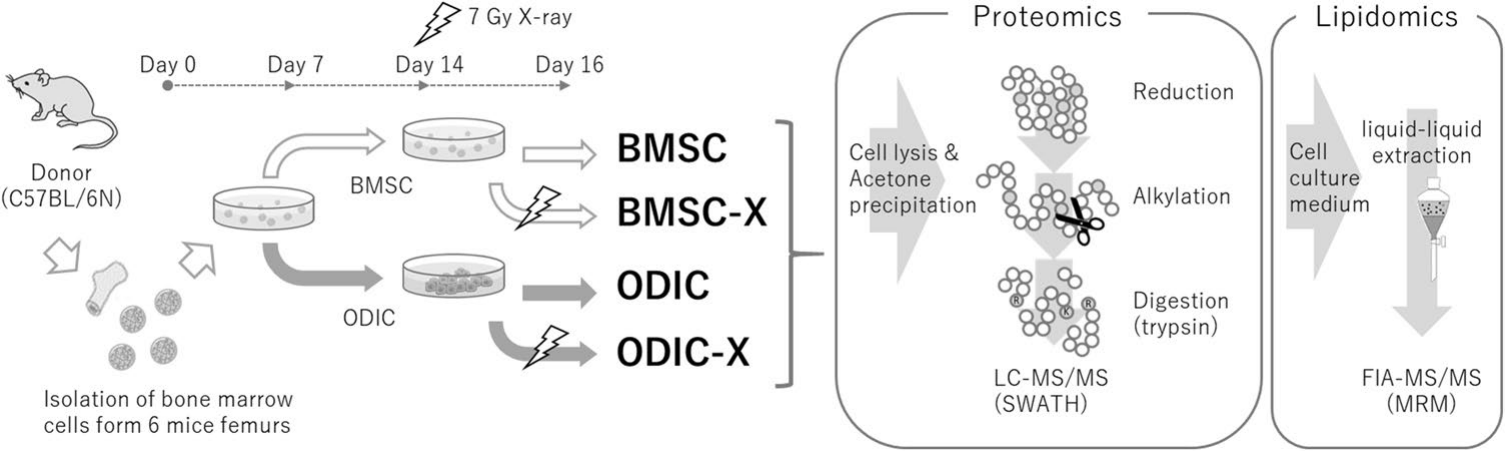
Experimental schematic for preparation of BMSC, ODIC, X-ray irradiated BMSC (BMSC-X), and X-ray irradiated ODIC (ODIC-X). The bone marrow cells were isolated from the mice femurs at day 0. And, these cells were cultured and induced to differentiate to osteoblast at day 7. On day 14, BMSCs and ODICs were irradiated with 7 Gy. The cell layers and cultured medium were collected at day 16 and prepared for proteomics and lipidomics, respectively.

**Figure 2:**
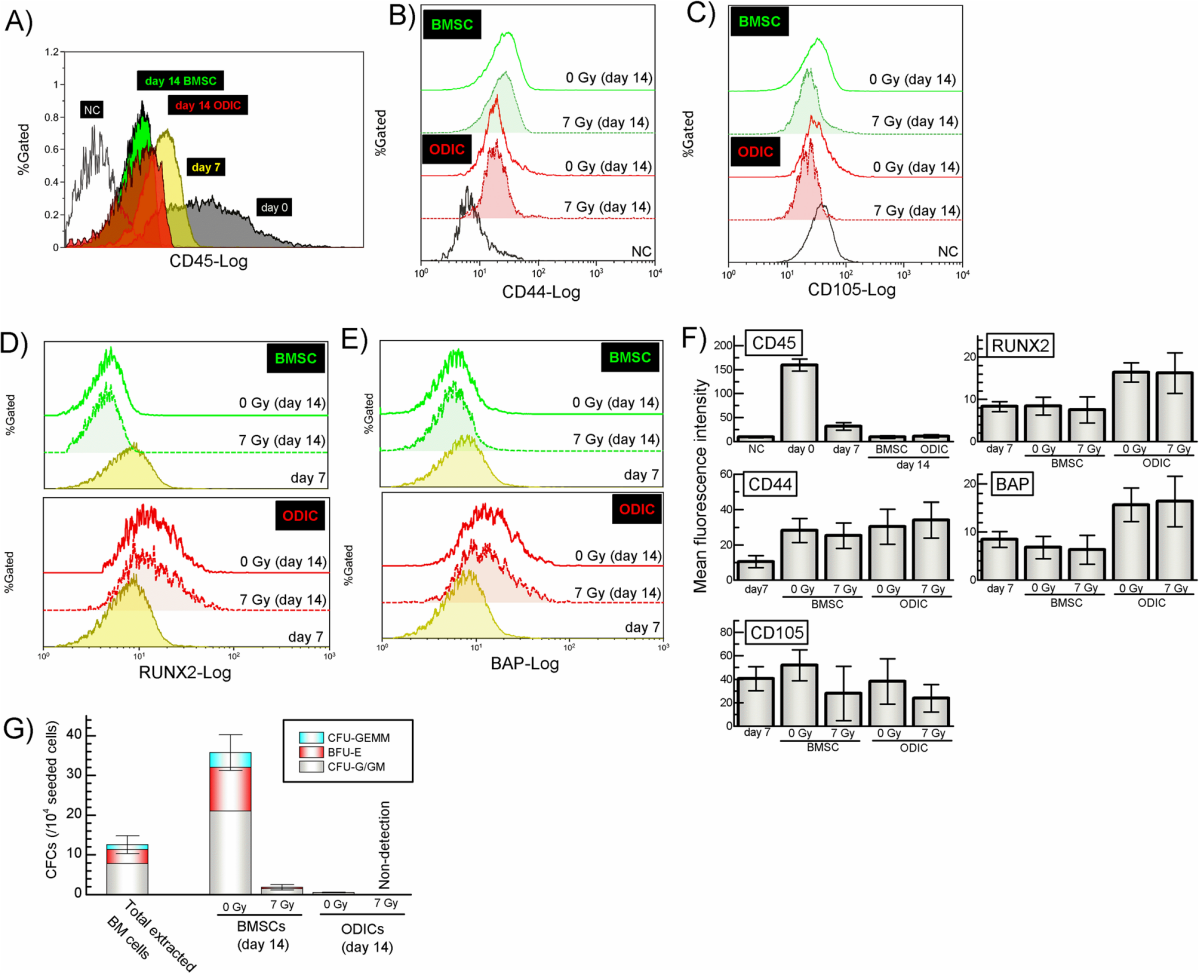
The osteoblastic differentiation level in mouse bone marrow cells. The expression of CD45 cell surface antigen on leukocyte and hematopoietic cells in bone marrow cell culture was analyzed as mean fluorescence intensity with flow cytometry (**A** and **F**). The expression of CD44 and CD105 cell surface antigen on stromal cell specific marker was also analyzed (**B**, **C** and **F**). The intracellular transcription factors (RUNX2 and BAP) that are osteoblast-specific differentiation markers were also analyzed (**D** and **E**). The hematopoietic clone potency of BMSC and ODIC was analyzed using colony formation assay (**G**). Each level of expression intensity was quantified by three mice lots. The values are mean ± S.E. of the mean of 3 separate experiments.

The experimental design is shown in Fig. 1. The bone marrow cells isolated from mice femurs were cultured in a standard medium containing 20% FBS for a week. Under these conditions, the blood cells were dropped out and BMSC survived on day 7 confirmed with flow cytometry measuring the hematopoietic cell marker CD45 (Fig. 2A). In addition, the stromal specific markers (CD44^+^CD45^-/low^CD105^-/low^) was also confirmed (Fig. 2B, C and F). The expression of osteoblast differentiation markers (RUNX2 and bone alkaline phosphatase (BAP)) in ODIC were compared between day 7 and day 14 with flow cytometry measuring (Fig. 2D, E and F). As further confirmation, it was confirmed that ODIC has extremely low potency of hematopoiesis (Fig. 2G). These markers were doubled 7 days after differentiation induction, indicating that osteoblasts were induced by differentiation treatment.

### Orthogonal partial least square-discriminant analysis (OPLS–DA) for class separation of proteome

**Figure 3:**
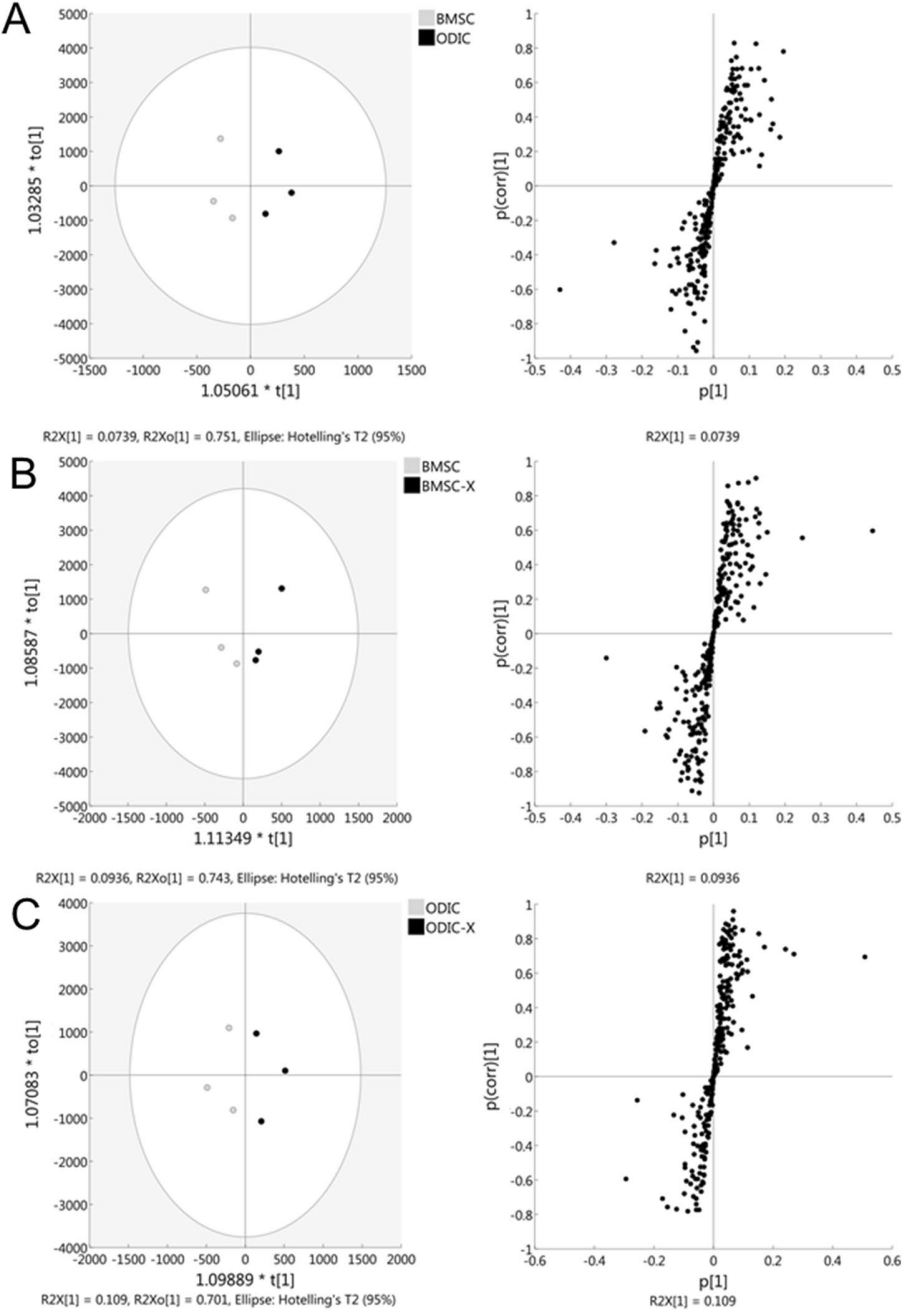
Multivariate analysis of proteomic profiles to compare impacts of X-ray radiation on BMSC and ODIC. OPLS-DA score plot, and loading S-plot are represented in the left, and right panels, respectively. (**A**) BMSC to ODIC: (**B**), BMSC to BMSC-X: (**C**), ODIC to ODIC-X. S-plot shows loading values of the first PLS component axis on the *p*_*1*_ axis and correlation loading values of the first PLS component axis on the *p*_*1*_*(corr)* axis from OPLS-DA.

Proteins extracted from whole cells of BMSC, BMSC-X, ODIC, and ODIC-X were measured in data-dependent acquisition mode, and 394 proteins were identified with 95% confidence. Of these, 321 protein-peptides met the criteria for SWATH measurement. Peak area values for peptides from each protein normalized by total peak area values were used for multivariate analysis OPLS-DA to visualize the class separation. The OPLS–DA model concentrates all the discriminating information into the first component. The score scatter plots of the OPLS-DA model in Fig. 3 left panels demonstrated satisfactory separation between BMSC and ODIC (Fig. 3A), between BMSC and BMSC-X (Fig. 3B), and between ODIC and ODIC-X (Fig. 3C) using one predictive component and one orthogonal component. The resultant S-plot of the developed OPLS-DA model between BMSC and ODIC identified 12 proteins that were highly correlated (|p(corr)|> 0.7) in the separation of the classes (Supplemental Table S2). In the same way, 40 proteins were identified with OPLS-DA that distinguish BMSC from BMSC-X (Supplemental Table S2). Several proteins including V-ATPases, Hsp90aa1, and CD44 overlapped with regulated proteins identified with OPLS-DA between ODIC and ODIC-X (44 proteins, Supplemental Table S2). Unlike in BMSC, the overlapping proteins were up-regulated in ODIC at 48 h after IR. V-ATPase is involved in protecting yeast cells against oxidative stress^18^. CD44 stabilizes xCT transporter to promote cystine uptake and suppresses the accumulation of ROS^19^. These proteins are considered to be involved in the stress response for ROS generated by IR, and we found a difference in that this response is activated in ODIC. Several additional proteins related to oxidative stress response such as AKR1A1, HMOX1, and SQSTM1 were increased only in ODIC. These results suggest that those proteins that showed the different quantitative changes may be related to different proteomic responses of BMSC and ODIC after IR.

### Ingenuity pathway analysis (IPA)

The up- and down-regulated proteins identified with OPLS-DA between irradiated groups and control groups were applied to the enrichment analysis with IPA canonical pathway analysis. The results of BMSC affected by IR showed 14 canonical pathways with *p*-value < 0.01 (Table 2). On the other hand, 29 pathways were enriched with *p*-value < 0.01 in ODIC (Table 3). BMSC and ODIC showed a common biological process that was affected by IR, e.g., iron homeostasis signaling pathway and RhoGDI signaling, but the regulations after IR were the opposite.

**Table 2:** Enrichment analysis of canonical pathway in IPA for the irradiated BMSC.

Ingenuity canonical pathways	− log(*p*-value)	Ratio	Molecules
Regulation of actin-based motility by rho	4.65	0.0426	CFL1, MYL12A, MYL6, PFN1
RhoA signaling	4.19	0.0325	CFL1, MYL12A, MYL6, PFN1
Semaphorin neuronal repulsive signaling pathway	4.10	0.0308	CD44, CFL1, MYL12A, MYL6
Phagosome maturation	3.85	0.0265	ATP6V0A1, ATP6V0D1, ATP6V1A, RAB5C
RhoGDI signaling	3.56	0.0222	CD44, CFL1, MYL12A, MYL6
Actin cytoskeleton signaling	3.24	0.0183	CFL1, MYL12A, MYL6, PFN1
PAK signaling	3.18	0.0309	CFL1, MYL12A, MYL6
Iron homeostasis signaling pathway	2.74	0.0219	ATP6V0A1, ATP6V0D1, ATP6V1A
Estrogen receptor signaling	2.59	0.0122	CFL1, HSP90AA1, MYL12A, MYL6
HOTAIR regulatory pathway	2.55	0.0187	CD44, COL1A2, LAMTOR5
Glucocorticoid receptor signaling	2.55	0.0119	HSP90AA1, KRT6B, KRT77, KRT79
Cdc42 signaling	2.50	0.0180	CFL1, MYL12A, MYL6
Signaling by rho family GTPases	2.05	0.0123	CFL1,MYL12A,MYL6
Axonal guidance signaling	2.00	0.0083	CFL1,MYL12A,MYL6,PFN1

**Table 3:** Enrichment analysis of canonical pathway in IPA for the irradiated ODIC.

Ingenuity canonical pathways	− log(*p*-value)	Ratio	Molecules
Iron homeostasis signaling pathway	3.89	0.0292	ATP6V0D1,ATP6V1A,HMOX1,LRP1
Remodeling of epithelial adherens junctions	3.54	0.0441	ACTG2,TUBB,VCL
RhoGDI signaling	3.43	0.0222	ACTG2,CD44,GNAS,MYL12A
Nrf2-mediated oxidative stress response	3.35	0.0212	ACTG2,AKR1A1,HMOX1,SQSTM1
Glycolysis I	2.96	0.0769	GAPDH,PGAM1
Gluconeogenesis I	2.96	0.0769	GAPDH,PGAM1
GP6 signaling pathway	2.83	0.0252	COL1A2,COL4A1,FGG
Cellular effects of sildenafil (Viagra)	2.71	0.0229	ACTG2,GNAS,MYL12A
Androgen signaling	2.66	0.0221	GNAS,HSP90AA1,HSPA4
Intrinsic prothrombin activation pathway	2.54	0.0476	COL1A2,FGG
Phagosome maturation	2.53	0.0199	ATP6V0D1,ATP6V1A,TUBB
Epithelial adherens junction signaling	2.53	0.0197	ACTG2,TUBB,VCL
BAG2 signaling pathway	2.52	0.0465	HSP90AA1,HSPA4
Aldosterone signaling in epithelial cells	2.48	0.019	HSP90AA1,HSPA4,Sacs
eNOS Signaling	2.47	0.0189	GNAS,HSP90AA1,HSPA4
Estrogen receptor signaling	2.47	0.0122	DDX5,GNAS,HSP90AA1,MYL12A
PFKFB4 signaling pathway	2.46	0.0435	GNAS,TKT
Glucocorticoid receptor signaling	2.44	0.0119	FGG,HSP90AA1,HSPA4,KRT1
Germ cell-sertoli cell junction signaling	2.38	0.0175	ACTG2,TUBB,VCL
Sertoli cell-sertoli cell junction signaling	2.29	0.0162	ACTG2,TUBB,VCL
Diphthamide biosynthesis	2.25	0.3330	EEF2
NADH repair	2.25	0.3330	GAPDH
D-glucuronate Degradation I	2.25	0.3330	AKR1A1
Leukocyte extravasation signaling	2.21	0.0152	ACTG2,CD44,VCL
Gap junction signaling	2.21	0.0152	ACTG2,GNAS,TUBB
HIF1α signaling	2.16	0.0146	HMOX1,HSP90AA1,HSPA4
Heme degradation	2.12	0.2500	HMOX1
Integrin signaling	2.12	0.0141	ACTG2,MYL12A,VCL
Actin cytoskeleton signaling	2.09	0.0138	ACTG2,MYL12A,VCL

**Figure 4:**
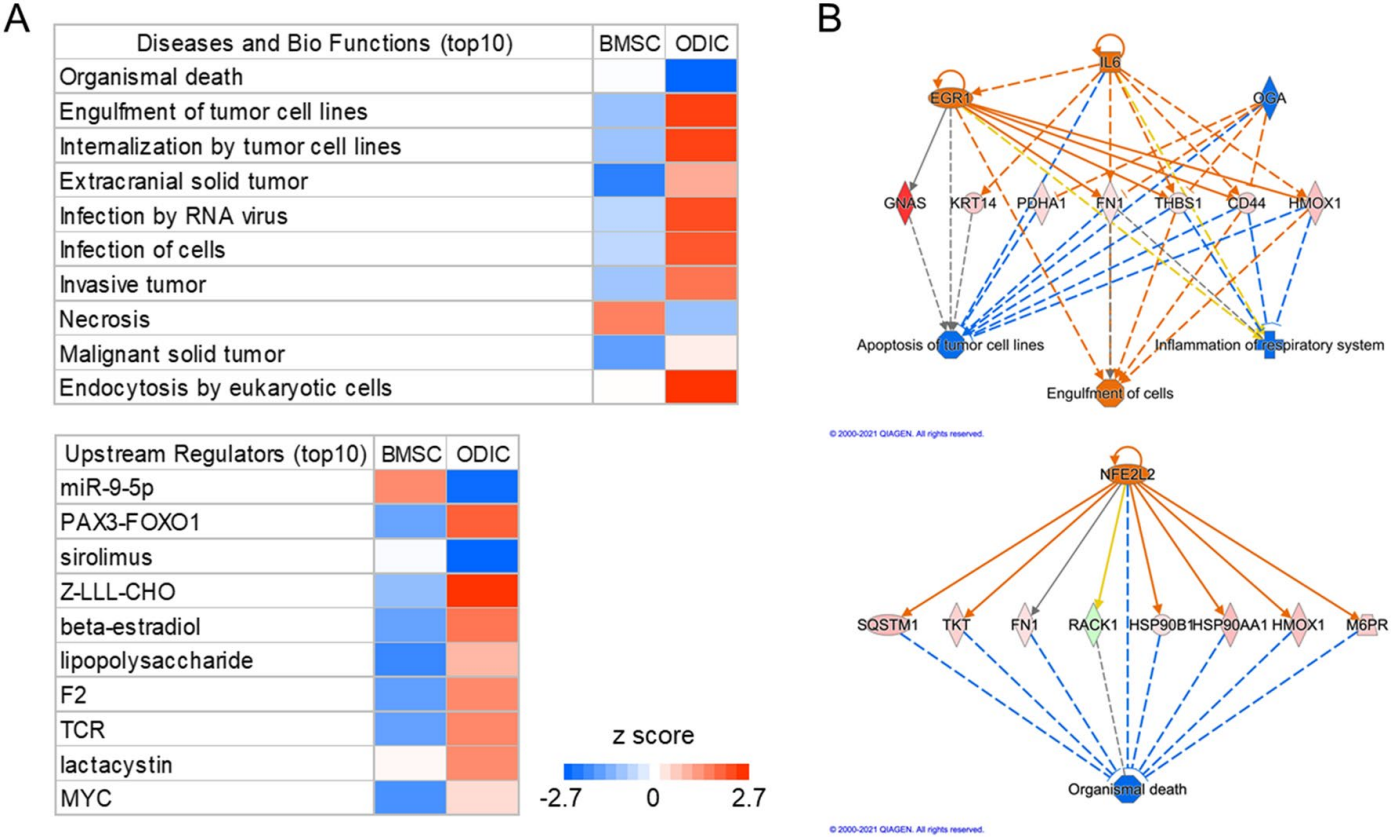
Annotation of proteome change by IR in BMSC and ODIC using IPA. (**A**), IPA comparison analysis between altered proteins in BMSC and ODIC after IR in terms of “Diseases and Bio Functions (upper panel)” and “Upstream Regulators (lower panel).” The up-regulated and down-regulated categories comparing irradiated with control groups are represented in blue and red, respectively. (**B**), Prediction of regulator effects correlated to the proteome change of ODIC after IR. The identified regulators of IL6 and Nrf2 (NFE2L2) are represented in the upper and lower panel, respectively. Red nodes indicate up-regulated proteins, while green nodes represent down-regulated proteins.

Comparison analysis by IPA was performed, and it was impressive that the changes in proteins involved in tumorigenesis were inversely regulated between BMSC and ODIC (Fig. 4A). This suggests that IR promotes tumorigenesis in ODIC. The prediction of upstream regulators showed opposite regulation of the MYC and beta-estradiol between BMSC and ODIC. Therefore, we used the IPA tool to predict regulator effects in ODIC based on the proteome altered by IR, and IL6 and Nrf2 were identified as regulators with Consistency Scores of 10.583 and 2.121, respectively (Fig. 4B). Proteins in the former pathway are mainly downstream factors of oncogene MYC, and upregulation of these proteins contributes to the inhibition of tumor cell apoptosis. AKR1A1, HMOX1, and SQSTM1 are under the control of the Nrf2 transcription factor, which plays an important role in the oxidative stress response, and was upregulated after IR. These results suggest that the Nrf2 pathway is activated in ODIC in response to ROS generated by IR.

### Lipidomics

**Figure 5:**
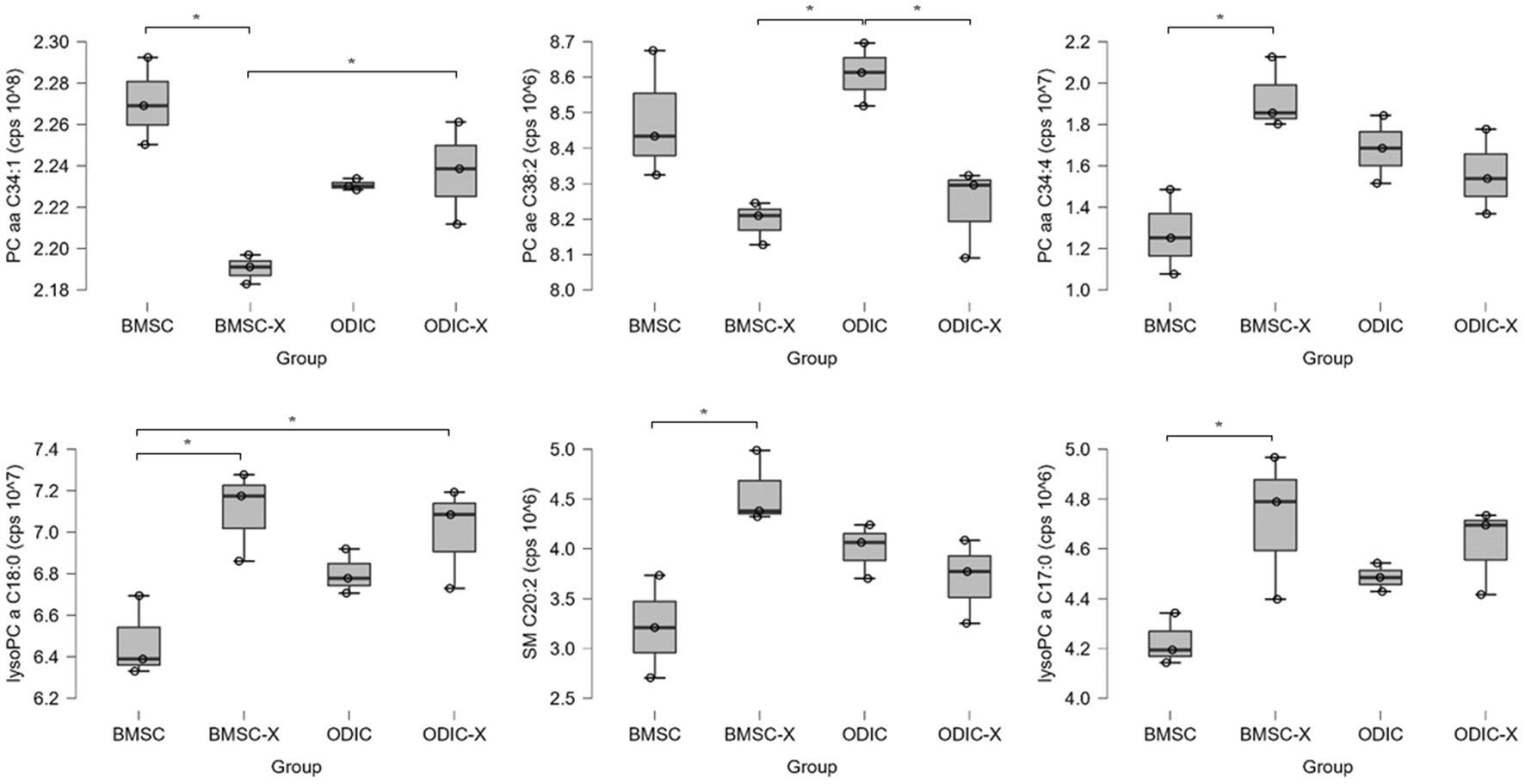
Relative quantities of secreted lipids showing a significant difference among BMSC, BMSC-X, ODIC, and ODIC-X. Peak areas of lipids are normalized to the total peak area, and statistical significance was determined by ANOVA, and lipids with significant differences (*p* < 0.05) are shown. Significant differences between the two groups were determined by post hoc test of Tukey’s HSD and are indicated by asterisks (*p_*tukey*_ < 0.05, **p_*tukey*_ < 0.01).

The lipidomics approach targeted lysophosphatidylcholine (lysoPC a), acyl-acyl phosphatidylcholine (PC aa), phosphatidylcholine with acyl-alkyl residue sum (PC ae), and sphingomyelin (SM). Semi-quantitative value of PC aa C34:1 secreted from BMSC was significantly lower than that of BMSC-X (Fig. 5 and Supplemental Table S3). On the other hand, PC aa C34:4, lysoPC a C18:0, SM C20:2, and lysoPC a C17:0 secreted from BMSC were significantly higher than those from BMSC-X. These lipids could be candidate biomarkers for detecting radiation-induced effects on BMSCs. The secreted lipid that was significantly altered by IR in ODIC was PC ae C38:2. In BMSCs, this lipid showed a tendency to decrease with irradiation. These results suggest that irradiation-induced changes in secreted lipids are greater in BMSCs than in ODICs.

SMs consist of ceramide and a phosphocholine or phosphoethanolamine head group. They are mainly found in cell membranes and thus influence cell signaling. Abnormal sphingolipid levels are associated with metabolic diseases. The increase in SM C20:2, a long to very long chain fatty acid SM, upon IR of BMSC suggests that cell membrane damage by oxidative stress was occurring^20,21^. However, no significant differences were observed in other SMs ranging from C16 to C26. Neither sum of long-chain (SM C14-18:x + SM (OH) C14-16:x) nor very long-chain fatty acid SMs (SM C22-26:x + SM (OH) C22-24:x) showed significant differences in any of the groups tested. Thus, SM C20:2 may be specifically released from the membrane of BMSCs upon IR. LysoPCs are derived from PCs via partial hydrolysis by phospholipase activity. Long-chain fatty acid lysoPCs have been linked with oxidative stress, mitochondrial dysfunction, and insulin resistance^20,21^. The increased lysoPCs in BMSC-X (lysoPC a C18:0 and lysoPC a C17:0) may be the result of oxidative stress caused by reactive oxygen species generated by IR. However, the lysoPCs are in part derived from the FBS in the medium, and uptake of such lysolipids from the medium is not considered in this study. Therefore, the increase in the secretion of lysoPCs by IR may partially involve a decrease in lipid uptake from FBS in the medium.

## Discussion

Given the lack of specific biomarkers indicative of bone marrow necrosis induced by ARS, the purpose of our study was to establish a targeted metabolomics platform to discover correlations between this disorder and molecular changes detectable in the secretome. The stromal cells act as feeder cells for the hematopoietic stem cell niche and its secretome would regulate hematopoietic stem cells^22,23^. The endosteal osteoblastic niche is known to be involved in the quiescent of hematopoietic stem cells^17,24^. So, we sought to elucidate the impact of IR on the lipidome of the niche cells with BMSC and ODIC. Such an approach is likely to be critical for designing stem cell-targeted therapies.

Radiation-induced H• or hydrated electrons react with dissolved oxygen molecules in water to produce O_2_^-^. In addition, H_2_O_2_ is formed by the reaction between OH• produced by irradiation and the reaction between OH• and OH^-^. OH• is formed from H_2_O_2_ by the Fenton reaction. When HO• is reduced to H_2_O, H• is removed from biomolecules such as lipids. This is the initial reaction of lipid radical oxidation, from which radical chain reactions are initiated. Arachidonic acid, an inflammatory lipid mediator, is known to be produced by the enzymatically degradation of phospholipid membranes by phospholipase A2 (PLA2)^25^. Although there is a report on radiation-induced oxidized lipid mediators in mouse serum, there has been little study of the MSC secretome targeting lipids. That study reported an increase in arachidonic acid-derived omega-6 unsaturated fatty acids and decrease of omega-3 oxylipins in the serum of mice exposed to 8 Gy of γ-ray^26^. In the present study, five discriminating lipids were identified in BMSC exposed to an X-ray dose of 7 Gy, which induced strong ARS, and could therefore be regarded as a potential set of predictive biomarkers of ARS: PC aa C34:1, PC aa C34:4, lysoPC a C18:0, SM C20:2, and lysoPC a C17:0 (Fig. 5). The lysoPC a C18:0 is an indicator of PLA2 activity hydrolyzing PC aa C38:4 into lysoPC a C18:0 and arachidonic acid, a polyunsaturated omega-6 fatty acid 20:4^27^. Although there have been no previous reports showing that PC aa C34:1 and PC aa C34:4 are associated with lipid mediator production, the possible link between the listed PCs and ARS is intriguing. The ability of MSCs to modulate cells involved in innate immunity and adaptive responses makes them a potential therapeutic tool for diseases related to immunity and inflammation^28^. The immunomodulatory functions of MSCs occur through direct cell-to-cell interaction or by secreting a broad range of factors including cytokines, growth factors, and prostaglandins^29^. Prostaglandin F2α, a metabolite of arachidonic acid, has been reported to diminish the phosphorylation of NF-κB leading to decreasing of IL-1β and granulocyte/macrophage colony-stimulating factor production in MSCs^30^. As discussed below, this study predicts NF-κB activation in ODIC based on proteome analysis, but could not reveal an association with lipid markers. It is a future issue to correlate the proteomic insights with lipidomics data based on biological pathways.

PC acts as a pool of many lipid messengers and can be a source of bioactive lipids such as phosphatidic acid, diacylglycerol, and lysoPC. PC is the main source of choline in the body, and itself or a mixture of derivatives form cell signaling molecules such as acetylcholine, platelet activator, and sphingophosphorylcholine. The fatty acid residues of PC are transferred to cholesterol by lecithin cholesterol acyltransferase to produce cholesterol esters. Accordingly, determining circulating levels of phospholipids and lipoproteins is considered important for diagnosing diseases associated with lipid transport^31^. Since the lipids targeted in this study can also be detected in humans, it will be of interest to examine the relationship between lipid biomarkers and human peripheral blood. Thus, the changes of lipid composition suggest a possible prophylactic or therapeutic approach based on the modulation of PC biosynthesis.

Some previous reports are investigating the plasma proteome analysis of the whole-body exposure of mice to a low dose of IR^32,33^. However, because of the abundance of major proteins in plasma, it is difficult to identify proteins that would reflect the response of the cells that form a stem cell niche to irradiation. Therefore, a strategy to analyze the effects of IR on BMSC and ODIC and then identify biomarker candidates to assess IR doses to individuals, as in this study, would be effective. In the present proteomics study, IL6 and Nrf2 were activated in ODIC after IR exposure, and the opposite regulation in BMSC (Fig. 4B). Rithidech et al*.* showed that the NF-κB and MAPKs pathways in hematopoietic stem cells play an essential role in the response to acute whole-body exposure to a low dose^34^. IL6 expression is induced by NF-κB^35^, suggesting that the NF-κB pathway in ODIC is activated even at a lethal dose of IR exposure. Upregulation of beta-estradiol was also identified as an upstream regulator in ODIC-X (Fig. 4A). Almeida et al*.* reported that stimulation of oxidative stress regulates the release of sex steroids as a counter during osteoblast induction^36^. Sex steroids cause proliferation and activation of some tumor cells^37,38^. Taken together, ODICs secrete sex steroids and cytokines and are active in cell proliferation, which may account for their markedly activated response to IR exposure.

Nrf2 is a master transcription factor regulating diverse genes in response to oxidative and/or electrophilic stresses^39,40^. The activation of the Nrf2 pathway in ODIC-X may be attributed to the production of ROS by IR. However, no evidence of Nrf2 activation was found in BMSC. Kato et al*.* reported the relationship between radio sensitivity and Nrf2-regulated gene expression using human placental/umbilical cord blood hematopoietic stem cells and showed that the number of colony-forming cells was significantly decreased and the multiple antioxidant genes were up-regulated after X-ray irradiated at a dose of 2 Gy^41^. These indicate that hematopoietic and nonhematopoietic stem cells respond differently to IR and may provide important insights into the effects on the designing stem cell-targeted therapies.

Collectively, the observed shift in PCs and SM distribution is a novel finding that needs to be further investigated mechanistically to understand if it has a causal relationship with the functional response shown by the proteomics analysis. Furthermore, since the lipids analyzed in this study are also detected in blood, it is an issue for future research to determine whether such a shift in lipid distribution can be observed in the peripheral blood of whole-body irradiated mice. A limitation of this study is the lack of coverage of lipids in lipidomics. The sample pretreatment was limited to lipids that were transferred to the organic solvent layer by liquid–liquid extraction. In addition, since the measured data were obtained in relative quantification and are dependent on the measurement environment, absolute quantification of biomarker candidates is necessary for future validation analysis.

## Materials and methods

### Materials

Alexa fluor® 488-conjugated anti-mouse RUNX2/CBFA1 polyclonal antibodies (pAb) and alexa fluor 488-conjugated anti-mouse BAP pAb were purchased from Novus Biologicals. Solvents for HPLC and lipid extraction including acetonitrile, water, formic, methanol, and dichloromethane acid were obtained from FUJIFILM Wako Pure Chemical Corporation. Reagents for protein chemistry including iodoacetamide, dithiothreitol, and trifluoroacetic acid were purchased from Merck.

### Animals

This study was approved by the President of Hirosaki University after the review by the Institutional Animal Care and Use Committee (Permit No. G12003). The animal experiments were performed in accordance with the national animal welfare guidelines and ARRIVE guidelines for reporting animal research^42^. Six male C57BL/6N mice were delivered at 7 weeks of age from the breeding facilities of Clea (Tokyo, Japan). Mice were housed in 3 mice/cage with paper chip bedding under specified pathogen-free conditions. All mice were housed in a conventional animal room with 12-h light/dark cycles, with food and water accessible ad libitum. The study used six normal weight (22–24 g) mice that had not been used in any other study and had no adverse events. To minimize distress in the mice, they were anesthetized with 2% isoflurane (Pfizer) and dissected after cervical spine fracture dislocation.

### Differentiation of BMSC and X-ray irradiation

Fresh bone marrow cells were isolated from 8-week-old mice femurs by flushing of PBS(-) with EDTA (day 0). These cells isolated from six mice were mixed (1.2 × 10^8^ cells) and seeded in 12 circle dishes (100 mmφ) with RPMI1640 supplemented with 20% fetal bovine serum (Gibco) and incubated for a week at 37 °C in a 5% CO_2_ atmosphere (1 × 10^7^ cells/dish). The 12 culture dishes were randomly assigned to the 4 groups. At this point (day 7), the hematopoietic cells were dropped out verified and adherent stromal cells with multipotency survived. To induce osteoblastic and bone differentiation, cells were cultured in a differentiation medium (20 mM β-glycerol phosphate disodium salt pentahydrate, 50 µM L-ascorbic acid 2-phosphate sesquimagnesium salt hydrate, and 100 nM dexamethasone) for a week (day 14). Cells were exposed to lethal doses (7 Gy) of X-rays at a dose rate of 1.0 Gy/min (150 kVp, 20 mA, 0.5 mm Al, and 0.3 mm Cu filters) using MBR-1520R-3 (Hitachi Medical Corp., Tokyo, Japan)^43^. After incubation for 48 h (day 16), the cell culture supernatants were collected for lipidomics analysis and stored at − 20 °C. The residual cells were harvested for proteomics analysis. The harvested cell pellet was suspended in 50 μl of 2% sodium dodecyl sulfate and 7 M urea, and the protein fraction was precipitated with acetone precipitation. All assays were carried out in technical triplicate and are represented as the mean of three independent experiments.

### Flow cytometry

The CD44, CD45 and CD105 cell surface antigen expressed on bone marrow cells was evaluated by flow cytometry (FACS Aria SORP, BD Biosciences, Franklin Lakes). The analysis software BD FACSDiva v.8.0.1 (BD Biosciences) was used. Samples containing 2 × 10^5^ cells were incubated under saturated concentrations (500 ng/ml) of the relevant phycoerythrin-cyanin-5-forochrome tandem (PC5) conjugated antimouse CD44, CD45, or CD105 monoclonal antibody (mAb) for 30 min at 4 °C, followed by washing. After removing erythrocytes in these bone marrow cells using RBC lysis buffer (eBioscience Inc., Affymetrix), flow cytometry analysis was performed. Intracellular transcription factor RUNX2/CBFA1 and BAP pAb were also analyzed using Fixation/Permeabilization Solution (BD Biosciences). Isotype‑matched mAb or pAb were used as negative controls.

### Clonogenic potency assay for hematopoiesis

The clonogenic potency of hematopoietic cell included BMSC or ODIC population was analyzed using colony formation assay. Colony-forming cells (CFCs), including colony-forming unit–granulocyte/granulo-macrophage (CFU-G/GM), burst-forming unit–erythroid (BFU-E) and colony-forming unit–granulocyte/erythroid/macrophage/megakaryocyte (CFU-GEMM) cells, were assayed by the methylcellulose method using MethoCult™ (STEMCELL Technologies Inc., Tokyo, Japan). The extracted or cultured mouse bone marrow cell were poured into each well of a 24-well plate with 300 μl of culture medium containing 100 ng/ml of recombinant IL-3, 100 ng/ml of recombinant SCF, 100 ng/ml of IL-6, G-CSF (10 ng/ml), EPO (4 U/ml), penicillin (100 U/ml) and streptomycin (100 μg/ml). Each plate was incubated at 37 °C in a humidified atmosphere containing 5% CO2 for 7 days. Colonies containing more than 50 cells were counted under 4× magnification using an inverted microscope (Olympus, Tokyo, Japan). After benzidine staining, blue and colorless colonies were scored as BFU-E and CFU-G/GM, respectively.

### Proteomics

The precipitated proteins of whole cells from one culture dish were resuspended in 50% trifluoroethanol, and reduced with 10 mM DTT. Free cysteine residues were alkylated with 20 mM iodoacetamide for 60 min at room temperature in the dark, and the remaining iodoacetamide was quenched by adding 10 mM DTT. The samples were then diluted with 100 mM ammonium bicarbonate. The samples were incubated with trypsin (AB Sciex) at 37 °C for 18 h. The samples were desalted with MonoSpin C18 (GL Sciences). The desalted peptides were analyzed with liquid chromatography (LC)-MS/MS using a nanoLC Eksigent 400 system (AB Sciex), coupled online to a TripleTOF6600 mass spectrometer (AB Sciex). Peptide separation was performed using LC on a nano C18 reverse-phase capillary tip column (75 μm × 125 mm, 3 μm, Nikkyo Technos CO). Acquired spectra were searched against the UniProt reviewed database using ProteinPilot 5.0.1 software (AB Sciex). Identifications were considered positive when identified proteins and peptides reached a 1% local FDR. The resulting group file was loaded into Peakview (v2.2.0, AB Sciex), and peaks from SWATH runs were extracted with a peptide confidence threshold of 99% and an FDR < 1%. Peak areas of individual peptides were normalized to the sum of peak areas of all detected peptides. After confirming that there are no outliers in the samples by principal component analysis, statistics of OPLS-DA were performed using Simca software (Infocom Corp). Pareto scaling was applied to the peak area values acquired by SWATH before the analyses. The core analysis of the IPA (Qiagen) was performed to interpret the proteome data in the context of biological processes, pathways, and networks.

### Lipidomics

Secreted lipids in the culture medium of one culture dish were measured with multiple reaction monitoring with the transitions of lysoPC with acyl residues, PC aa, PC ae, and SM (Table 1). Lipids in the culture medium were extracted with liquid–liquid extraction as follows. The cell culture medium (125 µl) was added to 875 µl water in a new glass screw-cap tube and incubated on ice for 10 min. Methanol (2.0 ml) and dichloromethane (0.9 ml) were added to the tubes. The tubes were mixed with vortexing, and then 1.0 ml water and 0.9 ml dichloromethane were added. The tubes were inverted 10 times and centrifuged at 1500 g for 10 min. The lower layer was collected and transferred to a fresh glass tube. The solvent was evaporated, and the residue was re-suspended in 200 μl methanol containing 0.1% formic acid.

The LC–MS/MS system was comprised of a high-performance liquid chromatography system (ExionLC AD, AB SCIEX) coupled to a QTRAP6500 + MS (AB Sciex) in electrospray ionization mode. Twenty microliters of the sample extract were used in the flow injection analysis in positive mode to capture glycerophospholipids and sphingolipids. All injections were carried out using methanol containing 0.1% formic acid with a flow rate setting of 0.2 ml/min. All metabolites were identified and quantified with multiple reaction monitoring with individual transition and parameters (Supplemental Table S1). For semi-quantification, peak areas of individual lipids were normalized to the sum of peak areas of all detected lipids.

## Supplementary Information

Supplementary Information 1.

Supplementary Information 2.

Supplementary Information 3.

## Data Availability

The proteomic data are available online using accession number “PXD025953’’ for Proteome Xchange and accession number “JPST001169” for jPOST Repository.
